# Evaluation of INSeq To Identify Genes Essential for *Pseudomonas aeruginosa* PGPR2 Corn Root Colonization

**DOI:** 10.1534/g3.118.200928

**Published:** 2019-01-31

**Authors:** Ramamoorthy Sivakumar, Jothi Ranjani, Udayakumar S. Vishnu, Sathyanarayanan Jayashree, Gabriel L. Lozano, Jessica Miles, Nichole A. Broderick, Changhui Guan, Paramasamy Gunasekaran, Jo Handelsman, Jeyaprakash Rajendhran

**Affiliations:** * Department of Genetics, School of Biological Sciences, Madurai Kamaraj University, Madurai, India; † Department of Molecular, Cellular and Developmental Biology, Yale University, New Haven, CT; ‡ Department of Molecular and Cell Biology, University of Connecticut, Storrs, CT,; § The Jackson Laboratory, Farmington, CT; ** VIT Bhopal University, Sahore, India; †† Wisconsin Institute for Discovery and Department of Plant Pathology, University of Wisconsin, Madison, WI 53715

**Keywords:** Plant-growth promoting rhizobacteria, INSeq, Tn-Seq, transposon mutagenesis, next generation sequencing, symbiosis

## Abstract

The reciprocal interaction between rhizosphere bacteria and their plant hosts results in a complex battery of genetic and physiological responses. In this study, we used insertion sequencing (INSeq) to reveal the genetic determinants responsible for the fitness of *Pseudomonas aeruginosa* PGPR2 during root colonization. We generated a random transposon mutant library of *Pseudomonas aeruginosa* PGPR2 comprising 39,500 unique insertions and identified genes required for growth in culture and on corn roots. A total of 108 genes were identified as contributing to the fitness of strain PGPR2 on roots. The importance in root colonization of four genes identified in the INSeq screen was verified by constructing deletion mutants in the genes and testing them for the ability to colonize corn roots singly or in competition with the wild type. All four mutants were affected in corn root colonization, displaying 5- to 100-fold reductions in populations in single inoculations, and all were outcompeted by the wild type by almost 100-fold after seven days on corn roots in mixed inoculations of the wild type and mutant. The genes identified in the screen had homology to genes involved in amino acid catabolism, stress adaptation, detoxification, signal transduction, and transport. INSeq technology proved a successful tool to identify fitness factors in *P*. *aeruginosa* PGPR2 for root colonization.

The rhizosphere is a dynamic, nutrient-rich environment on and around roots that supports intense activity among the resident soil microbiota and with the plant host. Root colonization is a complex process influenced by many factors. The primary bacterial traits important in root colonization are the ability of the bacterium to compete for niche space and respond to nutrients in the environment. This process involves sensing, response regulation, and chemotaxis toward the nutrient source (Broek and Vanderleyden 1995). In some soil microbes, such as *Pseudomonas* sp., initial adhesion of bacteria to the root surface has been shown to trigger the expression of cell density-regulated genes that shape community behavior such that there is a direct correlation between bacterial population density and seed colonization (Espinosa-Urgel and Ramos 2004). This coordinated gene expression enables the bacteria to establish as microcolonies (multicellular aggregates) and eventually leads to the formation of biofilm-like structures on the root surface. Previous work has identified other bacterial traits that contribute to root colonization by bacteria, such as synthesis of amino acids, uracil, and vitamin B1, site-specific recombinase Sss, NADH dehydrogenase, and a Type Three Secretion System (TTSS) (Lugtenberg and Kamilova 2009). Moreover, soil bacteria that positively influence the growth of plants, termed plant-growth-promoting rhizobacteria (PGPR), trigger a cascade of molecular signals that play a vital role in establishing a mutualistic relationship. Insight into these signaling processes is essential to understanding this complex relationship and improving such beneficial interactions for the benefit of agricultural production (Morrissey *et al.*, 2004).

Fluorescent pseudomonads are well-known for their beneficial associations with plants derived from their aggressive colonization of roots and production of antimicrobial compounds active against pathogens (de Weger *et al.*, 1986; Chin-A-Woeng *et al.*, 2000; Lugtenberg *et al.*, 2001). However, roles of plant root-derived compounds and the suites of genes involved in these beneficial associations have not been fully elucidated (Costa *et al.*, 2007; Hartmann *et al.*, 2009). In this study, we report the molecular determinants of *Pseudomonas aeruginosa* PGPR2 that are essential for colonization of corn roots. Although *P*. *aeruginosa* has often been reported as an opportunistic pathogen of humans, it is also found in association with plants and some strains promote plant growth (Anjaiah *et al.*, 2003; Walker *et al.*, 2004).

*P*. *aeruginosa* PGPR2 is an efficient root colonizer and promotes plant growth. Previously, we reported the antagonistic properties of this bacterium against *Macrophomina phaseolina*, a fungal pathogen of plants (Illakkiam *et al.*, 2013). Sequence analysis of the *P*. *aeruginosa* PGPR2 genome identified genes that might contribute to plant-growth promotion and disease suppression, including genes responsible for ACC deaminase, activation of auxin signaling, siderophore production, antifungal compound synthesis (*i.e.*, HCN, phenazines), and phosphate solubilization (Illakkiam *et al.*, 2014). However, whether these genes directly contribute to the proposed functions is not known.

Next-generation sequencing technology has opened a new area in functional genomics research. High throughput transposon insertion sequencing, such as HITS (Gawronski *et al.*, 2009), TraDIS (Langridge *et al.*, 2009), INSeq (Goodman *et al.*, 2009) and Tn-seq (van Opijnen *et al.*, 2009), has emerged as a powerful functional genomics tool that establishes causal relationships between genes and bacterial behavior. This strategy combines transposon mutagenesis and high-throughput sequencing, which allows simultaneous assessment of the fitness of thousands of discrete mutants for a particular function. Thus, INSeq enables identification of genetic elements required for the fitness of an organism *in vitro* or *in vivo*. Recently, Cole *et al.* (2017) reported *Pseudomonas simiae* genes required for *Arabidopsis thaliana* root colonization using randomly barcoded transposon mutagenesis sequencing (RB-TnSeq). In the study reported here, we employed INSeq analysis to unravel the genetic elements responsible for mutualistic interactions using corn and *P. aeruginosa* PGPR2 as a model system. We identified 108 genes that were essential for fitness of strain PGPR2 during corn root colonization.

## Materials and Methods

### Bacterial strains and growth conditions

The bacterial strains and plasmids used in this study are listed in Table 1. *Pseudomonas aeruginosa* PGPR2 and *Escherichia coli* were grown at 30° and 37°, respectively, and routinely sub-cultured in Luria Bertani (LB) medium. When required, the medium was solidified using 2% agar. Antibiotics were added as needed at the following concentration (unless otherwise specified): ampicillin, 100 µg ml^-1^; gentamicin, 40 µg ml^-1^; irgasan, 25 µg ml^-1^.

**Table 1:** Strains and plasmids used in this study

Bacterial strains / plasmids	Description*^a^*	Source or Reference
***P .aeruginosa***		
PGPR2	*P. aeruginosa* wild type strain	Illakkiam *et al.* (2013)
PGPR2 ΔTrpD	TrpD^-^ derivative of PGPR2	This study
PGPR2 ΔHom	Hom^-^ derivative of PGPR2	This study
PGPR2 ΔOprF	OprF^-^ derivative of PGPR2	This study
PGPR2 ΔCbrA	CbrA^-^ derivative of PGPR2	This study
***E. coli***		
DH5α	F− Ø80lacZ M15 endA recA hsdR(rk−mk−) supE thi gyrA relAΔ(lacZYA-argF) U169	Laboratory Stock
S17-1λ-pir	recA pro hsdR RP4-2-Tc::Mu-Km::Tn7λ-pir	de Lorenzo *et al.* (1990)
**Plasmids**		
pIVETP	Tc^R^, Ap^R^, *ori* R6K, gene replacement vector	Rainey (1999)
pGEM-T	Ap^R^, PCR product cloning vector	Promega
pUCP24	Gm^R^, *E. coli*—*Pseudomonas* Shuttle vector	West *et al.* (1994)
pSAM-BT	Ap^R^, ermG^R^, *ori* R6K, transposon integration vector with mariner transposase *himar1c9*	Goodman *et al.* (2009)
pBT20	Gm^R^, mini transposon vector with Himar-1 mariner transposase	Kulasekara *et al.* (2005)
pSAM_BT20	Ap^R^, Gm^R^, transposon integration vector	This study
pTrpD1	Ap^R^, 600-bp deletion construct of *trpD* gene in pGEM-T	This study
pTrpD2	Ap^R^, Gm^R^, Insertion of gentamicin cassette into 600-bp deletion construct of *trpD* gene in pTrpD1 [Δ*trpD*:: Gm^R^]	This study
pTrpD3	Tc^R^, Ap^R^, Gm^R^, 1.35-kb deletion construct of *trpD* gene subcloned into *XhoI* site of pIVETP	This study
pHom1	Ap^R^, 600-bp deletion construct of *hom* gene in pGEM-T	This study
pHom2	Ap^R^, Gm^R^, Insertion of gentamicin cassette into 600-bp deletion construct of *hom* gene in pTrpD1 [Δ*hom*:: Gm^R^]	This study
pHom3	Tc^R^, Ap^R^, Gm^R^, 1.35-kb deletion construct of *hom* gene subcloned into *XhoI* site of pIVETP	This study
pOprF1	Ap^R^, 600-bp deletion construct of *oprF* gene in pGEM-T	This study
pOprF2	Ap^R^, Gm^R^, Insertion of gentamicin cassette into 600-bp deletion construct of *oprF* gene in pTrpD1 [Δ*oprF*:: Gm^R^]	This study
pOprF3	Tc^R^, Ap^R^, Gm^R^, 1.35-kb deletion construct of *oprF* gene subcloned into *XhoI* site of pIVETP	This study
pCbrA1	Ap^R^, 1.2-kb deletion construct of *cbrA* gene in pGEM-T	This study
pCbrA2	Ap^R^, Gm^R^, Insertion of gentamicin cassette into 1.2-kb deletion construct of *cbrA* gene in pTrpD1 [Δ*cbrA*:: Gm^R^]	This study
pCbrA3	Tc^R^, Ap^R^, Gm^R^, 2.0-kb deletion construct of *cbrA* gene subcloned into *XhoI* site of pIVETP	This study

a Ap^R^, Tc^R^, Gm^R^ and ErmG^R^ resistance to ampicillin, tetracycline, gentamicin, and erythromycin respectively.

### Construction of pSAM-BT20 vector

The transposon delivery vector, pSAM-BT20, was derived from pSAM-BT (Goodman *et al.* 2009). The erythromycin resistance gene was removed with *Xho*I and *Xba*I (New England Biolabs, Ipswich, MA) and replaced with a gentamicin resistance gene that was PCR amplified with primers GenF-GenR (Table S1). The transposase gene was removed with *Bam*HI and *Not*I (New England BioLabs) and replaced with the transposase gene from pBT20, which was PCR amplified using primers Trans F-Trans R (Goodman *et al.*, 2009).

### Construction of transposon insertion mutant library

The transposon library was constructed by conjugation of *P*. *aeruginosa* PGPR2 with *E. coli* S17-1 λ *pir* harboring pSAM-BT followed by selection of exconjugants for gentamicin resistance. Briefly, the donor and recipient strains were grown separately overnight. Cultures were mixed and pelleted, then washed with fresh LB medium, suspended in 100 µl of LB medium and spotted on an LB agar plate, and incubated at 30° for 3 hr. The exconjugants containing insertions were selected by plating on LB medium supplemented with gentamicin (40 µg ml^-1^) and irgasan (25 µg ml^-1^) for counterselection against the donor *E. coli* strain. The plates were incubated at 30° for 24 hr. Successful integration of the transposon into the genome was verified by performing PCR for the gentamicin resistance gene using primers GenF and GenR. The colonies were pooled using sterile phosphate-buffered saline containing 15% glycerol. One ml aliquots of the mutant library suspension were placed in vials and stored at -80° until further use. One vial was retrieved from the stock and enumerated by standard dilution plating.

Southern blot hybridization was performed to confirm random integration and single-copy insertion of the transposon into the genome of PGPR2. Genomic DNA was isolated from 13 random mutants using the Qiagen blood and tissue mini kit according to the manufacturer’s instructions. Genomic DNA (1 µg) was digested with *Hin*dIII and separated by electrophoresis on a 0.8% Tris-boric acid-EDTA agarose gel and transferred to a positively charged nylon membrane. The gentamicin-resistance cassette was used as a probe and labeled using the Digoxigenin-dUTP (DIG) DNA labeling kit (Roche, Switzerland) according to the manufacturer’s instructions. Bound DNA was hybridized with a DIG-labeled gentamicin- resistance gene generated by random primer amplification and visualized by autoradiography.

We generated a library of 39,500 insertion mutants in which integration of transposons into the genome was confirmed by PCR amplification from the genomic DNA of 20 random mutants, which showed the presence of the gentamicin resistance cassette, but not the transposase gene (Fig. S1). Southern blotting showed that each PGPR2 mutant had only one unique transposon insertion in the genome, as only one fragment from each mutant hybridized to the probe. Also, the single integration was random, as indicated by the range of size of the fragments carrying the transposon (Fig. S2). On average, transposon integration was found every 169 bp in the genome. Fig. S3 depicts the random insertions throughout the genome.

### Plant system for INSeq selection

**Figure 1:**
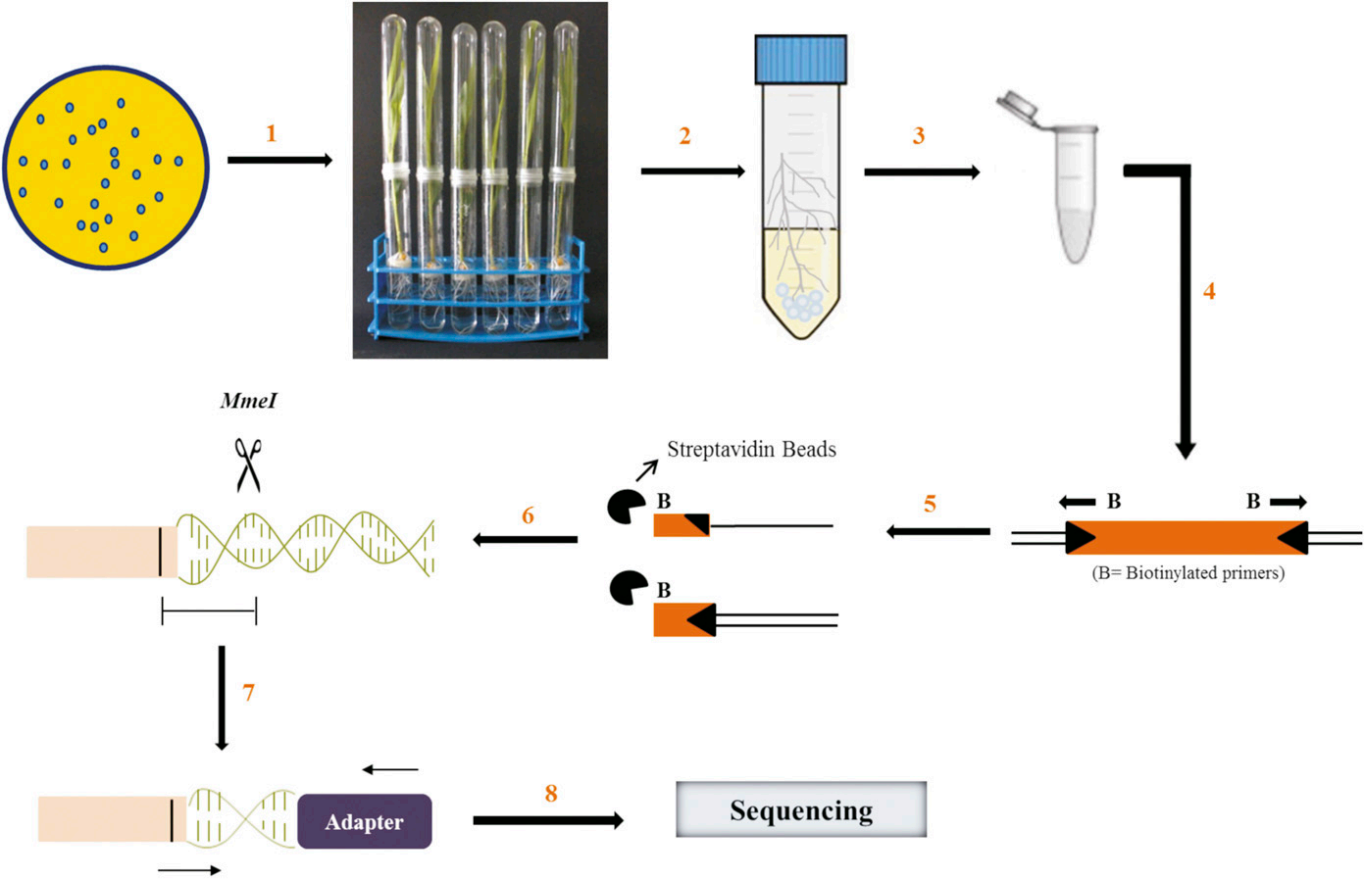
Workflow of insertion sequencing. A transposon insertion library in *P. aeruginosa* PGPR2 with ∼40,000 mutants was generated and a suspension of 4 X 10^6^ CFU ml^-1^ was applied to germinated corn seedlings (step 1). Seven days post inoculation, the roots were excised aseptically and briefly vortexed in 20 ml saline containing glass beads to detach the bacteria adhered to root surface (step 2). Genomic DNA was extracted and purified from both input and output populations (step 3). Linear PCR was performed using biotinylated primers to amplify transposon integration site (step 4). The amplified product was captured using streptavidin beads and the second DNA strand was synthesized (step 5). A DNA fragment was digested with *MmeI* enzyme and ligated with Illumina barcodes (step 6). The product was amplified for a restricted number of PCR cycles and ligated with Ion Torrent adapters on either side (step 7). The sequencing was performed using the Ion Torrent platform (step 8) (adapted from Goodman *et al.*, 2011).

Surface-disinfected corn seeds were germinated on moist filter paper for three days and transferred to the gnotobiotic hydroponic plant nutrient medium (Hoagland and Arnon 1950) without any carbon source supplementation. This medium does not support bacterial growth and therefore the growth of *P. aeruginosa* PGPR2 is entirely dependent on nutrients provided from the plant. An aliquot of the mutant library was thawed, pelleted, and washed three times with sterile 10 mM MgSO_4_. The suspension was diluted to ∼4 × 10^6^ CFU ml^-1^ and inoculated onto germinated seedlings in triplicate (n = 3). A portion of the suspension was used for genomic DNA isolation (input pool). The plants were maintained in the greenhouse with 16 hr light and 8 hr of dark. Seven days after inoculation, the roots were aseptically excised, washed gently to eliminate weakly adhered bacterial cells and transferred to 50-ml tubes containing 0.85% saline and ten glass beads (3 mm in diameter). The tubes were vortexed briefly to detach bacteria from the root surface, the root was discarded and the suspension was used for genomic DNA isolation (output pool). The workflow is shown in Figure 1.

### INSeq library preparation and sequencing

Total DNA was isolated from the input and output populations using QIAGEN DNeasy blood and tissue kit according to the manufacturer’s instructions. The extracted DNA was used as template to amplify transposon-insertion junctions with appropriate barcodes as previously described (Goodman *et al.*, 2011). The amplicons obtained were ligated with Ion Torrent adapters, and samples were pooled in equimolar concentration and sequenced using Ion Torrent PGM on a 318 chip.

### Data analysis

Adapters were trimmed from the raw reads using Cutadapt and the reads were split based on barcodes using FastX barcode splitter. The INSeq data were analyzed using the online software ESSENTIALS (http://bamics2.cmbi.ru.nl/websoftware/essentials) (Zomer *et al.*, 2012). The processed reads were mapped to the *P*. *aeruginosa* PGPR2 genome (Accession number ASQO01000001-ASQO01000198). The functional classification was done according to protein annotation by COG database using WebMGA (Wu *et al.*, 2011).

### Construction of P. aeruginosa PGPR2 deletion mutants

*P*. *aeruginosa* PGPR2 deletion mutants were generated in the *trpD*, *hom*, *oprF* and *cbrA* genes. Briefly, PCR primers were designed to amplify a partial fragment of each gene in the 5′ and 3′ regions and contained *Hind*III restriction sites (Table S1). The gentamicin-resistance cassette was amplified from the pUCP24 vector (Table 2) with primers containing *Hind*III restriction sites. Appropriate DNA fragments were then restricted and ligated with a gentamicin cassette resulting in deletions of each gene. This *in vitro* product was then cloned into the *Xho*I site of suicide vector pIVETP (Table 1), which was then mobilized into *P*. *aeruginosa* PGPR2 from *E*. *coli* S17-1 λ-*pir*. The exconjugants resulting from double homologous recombination were selected on LB agar containing gentamicin and irgasan. Deletion alleles within each mutant were confirmed by PCR.

**Table 2:** Functional categories of *P. aeruginosa* PGPR2 genes required for the fitness (at q < 0.05) during corn root colonization

Locus tag	Annotation	Fold Change of mutant*^a^*	q – Value
**Cytochrome biosynthesis**			
PGPR2_25200	cytochrome B	−4.5	0.005
PGPR2_07910	cytochrome C assembly protein	−4.1	0.04
PGPR2_20185	cytochrome oxidase subunit I	−3.0	0.02
**Motility and adhesion**			
PGPR2_20675	*flhF*- flagellar biosynthesis regulator	−8.1	0.001
PGPR2_22450	*flgD*- flagellar basal body rod modification protein	−3.7	0.02
PGPR2_17290	fimbrial subunit cupA4	−6.4	0.005
**Energy production**			
PGPR2_13755	*nuoH*- NADH:ubiquinone oxidoreductase subunit H	−10.4	0.0001
PGPR2_13780	*nuoB*- NADH dehydrogenase subunit B	−9.7	7.58E-07
PGPR2_13740	*nuoK*- NADH dehydrogenase subunit k	−9.7	8.12E-07
PGPR2_13770	*nuoE*- NADH dehydrogenase subunit E	−8.3	0.0008
PGPR2_13730	*nuoN*- NADH:ubiquinone oxidoreductase subunit N	−7.9	1.05E-05
PGPR2_13735	*nuoM*- NADH:ubiquinone oxidoreductase subunit M	−7.1	7.19E-05
PGPR2_13775	bifunctional NADH:ubiquinone oxidoreductase subunit C/D	−6.9	3.89E-06
PGPR2_13760	*nuoG*- NADH dehydrogenase subunit G	−4.4	0.008
**Carbon metabolism**			
PGPR2_13800	*aceA*- isocitrate lyase	−7.3	1.00E-05
PGPR2_04700	*glcB*- malate synthase	−6.8	8.12E-06
**Nitrogen assimilation**			
PGPR2_08360	*glnD*- PII uridylyl-transferase	−5.8	1.31E-05
**Metabolism of amino acids**			
PGPR2_05445	*trpD*- anthranilate phosphoribosyltransferase	−48	2.92E-13
PGPR2_07965	*hom*- homoserine dehydrogenase	−41	1.39E-08
PGPR2_27075	ketol-acid reductoisomerase	−25.7	3.21E-09
PGPR2_11060	*leuB*- 3-isopropylmalate dehydrogenase	−24.7	9.75E-09
PGPR2_28825	*gltB*- glutamate synthase subunit alpha	−24.3	5.50E-12
PGPR2_27085	*ilvL*- acetolactate synthase 3 catalytic subunit	−21.6	6.45E-07
PGPR2_04235	*metX*- homoserine O-acetyltransferase	−20.4	1.39E-08
PGPR2_05440	*trpG*- anthranilate synthase subunit II	−20.1	0.0001
PGPR2_05340	*trpE*- anthranilate synthase subunit I	−14.2	4.32E-08
PGPR2_29970	*argH*- argininosuccinate lyase	−13.4	3.18E-06
PGPR2_25290	*hisD*- histidinol dehydrogenase	−13.7	8.00E-06
PGPR2_11110	*metZ*- O-succinylhomoserine sulfhydrylase	−12.5	1.58E-05
PGPR2_29675	*argA*- N-acetylglutamate synthase	−12.5	9.10E-05
PGPR2_28820	*gltD*- glutamate synthase subunit beta	−11.5	1.39E-05
PGPR2_04445	*metF*- 5,10-methylenetetrahydrofolate reductase	−11.6	0.0002
PGPR2_11085	*trpF*- N-(5′-phosphoribosyl) anthranilate isomerase	−11.5	0.0003
PGPR2_09040	*argG*- argininosuccinate synthase	−10.6	0.0006
PGPR2_10940	aspartate aminotransferase	−10.0	0.0001
PGPR2_08980	*argF*- ornithine carbamoyltransferase	−7.2	0.0001
PGPR2_11045	*leuC*- isopropylmalate isomerase	−6.4	0.004
PGPR2_23380	*aruF*- arginine N-succinyltransferase	−6.1	0.007
PGPR2_09190	arogenate dehydratase	−5.1	0.007
PGPR2_04230	methionine biosynthesis protein MetW	−4.4	0.04
PGPR2_19145	*cysH*- phosphoadenosine phosphosulfate reductase	−4.3	0.009
PGPR2_18710	*cysI*- sulfite reductase	−3.5	0.01
**Metabolism of fatty acids**			
PGPR2_14775	acyl-CoA dehydrogenase	−13.5	0.0002
PGPR2_19190	enoyl-CoA hydratase	−3.7	0.005
**Metabolism of vitamins**			
PGPR2_20670	*cbiA*- cobyrinic acid a,c-diamide synthase	−25	1.09E-05
PGPR2_04190	*thiG*- thiazole synthase	−5.2	0.003
PGPR2_04790	*bioB*- biotin synthase	−4.8	0.005
PGPR2_05260	*pdxA*- 4-hydroxythreonine-4-phosphate dehydrogenase	−4.8	0.002
PGPR2_04395	adenosylmethionine-8-amino-7-oxononanoate aminotransferase	−2.9	0.02
**DNA replication, recombination, and repair**			
PGPR2_23005	*ruvB*- Holliday junction DNA helicase	−19	5.37E-06
PGPR2_11105	*purF*- amidophosphoribosyltransferase	−10.5	4.45E-05
PGPR2_07835	*purL*- phosphoribosylformylglycinamidine synthase	−10.5	0.0003
PGPR2_23120	*purM*- phosphoribosylaminoimidazole synthetase	−8.4	0.0004
PGPR2_13155	transposase ISPpu14	−5.6	0.0145
PGPR2_31725	*parA*- chromosome partitioning protein	−4.5	0.01
**Stress adaptation and detoxification**			
PGPR2_23995	*algU*- RNA polymerase sigma factor	−16.8	5.75E-05
PGPR2_29670	*gshA*- glutamate–cysteine ligase	−8.9	0.0006
PGPR2_23020	*pmpR*- hypothetical protein	−6.2	0.002
PGPR2_12560	glutathione S-transferase	−3.4	0.03
**Sensors and regulators**			
PGPR2_31765	*cbrA*- two-component sensor	−10.8	1.19E-05
PGPR2_27925	sensor histidine kinase RetS protein	−9.5	0.003
PGPR2_08335	ArsC family transcriptional regulator	−8.9	0.0007
PGPR2_29245	sensor histidine kinase	−6.3	0.001
PGPR2_11615	PasA sensor protein	−6.2	0.001
PGPR2_25045	two-component sensor	−4.6	0.04
PGPR2_12420	MarR family transcriptional regulator	−5.8	0.008
PGPR2_12765	transcriptional regulator	−3.8	0.03
**Transporters**			
PGPR2_19040	*oprF*- OmpF family protein	−35	2.48E-15
PGPR2_05250	magnesium transporter ApaG	−7.1	0.0001
PGPR2_29805	ABC transporter permease	−6.2	0.006
PGPR2_12725	VacJ ABC transporter	−5.0	0.0003
PGPR2_25020	AmpG permease	−4.1	0.03
PGPR2_25320	organic solvent ABC transporter substrate binding protein	−3.6	0.01
PGPR2_23050	*oprD*- porin	−3.3	0.008
PGPR2_29810	multidrug ABC transporter ATP-binding protein	−3.0	0.04
**Osmoregulation**			
PGPR2_29035	*mdoG*- glucan biosynthesis protein	−12.5	0.0006
PGPR2_29030	*mdoH*- glucosyltransferase	−9.2	1.05E-05
**Protein synthesis, folding and degradation**			
PGPR2_18895	*clpX*- ATP dependent protease	−14	1.19E-05
PGPR2_10340	*prc*- periplasmic tail-specific protease	−6.8	3.60E-05
**Unknown**			
PGPR2_22850	hypothetical protein	−12.4	1.09E-05
PGPR2_25425	hypothetical protein	−4.7	0.0007
PGPR2_30555	hypothetical protein	−4.2	0.03
PGPR2_06760	hypothetical protein	−3.3	0.01
PGPR2_19100	hypothetical protein	−2.6	0.03

a For each gene, fold changes were calculated by comparing the relative abundance of sequence reads in the input and output populations. Significantly, underrepresented genes were considered conditionally essential genes.

### Root colonization assay

To verify the reduction in colonizing ability of isogenic mutants constructed in *P. aeruginosa* PGPR2, root colonization assays were performed. Briefly, corn seeds were surface sterilized as above and germinated seedlings were transferred to gnotobiotic hydroponic medium. Corn plantlets grown in hydroponic system was inoculated with ∼4 × 10^6^ CFU of wild type and mutant strains. At 7 days post inoculation, the roots were aseptically excised and transferred to a 50 ml tube containing 20 ml 0.85% saline. The tube was vortexed for 1 min to detach adhered bacteria, and the suspension was enumerated by standard dilution plating. The experiment was repeated three times with five replicates. Differences were considered statistically significant when *P* < 0.001.

### Competitive root colonization

To test the competitive root-colonizing ability of mutant strains, 1:1 mixtures of the wild-type and each mutant strain were applied to plantlets grown in hydroponic medium as described above. After seven days, bacterial cells were isolated from the root surface and plated on LB medium supplemented with and without gentamicin to differentiate the mutant from the wild-type strain. A strain’s competitiveness was expressed as the percentage of total colony forming units recovered from corn roots represented by the strain. Each experiment was repeated three times with five replicates per treatment.

### Data availability statement

Mutant library and the mutant strains are available on request. Table S1 contains the list of primers used in this study. Table S2 contains the genes essential for the growth of *P*. *aeruginosa* PGPR2 in LB, and Table S3 contains the genes required for its fitness during root colonization. Supplemental material available at Figshare: https://doi.org/10.25387/g3.7627262.

## Results

### INSeq genetic analysis of root colonization by P. aeruginosa PGPR2

We generated and validated a library of approximately 39,500 insertion mutants in *P. aeruginosa* PGPR2, which contains 6,803 genes, providing approximately sixfold coverage or an average of six insertions per gene. To identify *P. aeruginosa* PGPR2 genes essential for the root colonization in corn, we inoculated each corn seedling with 4 × 10^6^ CFU of the insertion mutant library. The bacterial population increased 100-fold in seven days, after which the input and output populations were analyzed. A total of 993 genes were necessary for the growth of PGPR2 in LB medium, including genes required for normal growth, such as those encoding tRNA and rRNA (Table S2). Functional categorization revealed many of these essential genes are involved in translation (9%), transcription (8.3%), replication (7.8%), cell wall biogenesis (8.5%) and unknown functions (20.5%) (Fig. S4).

### Genes essential for P. aeruginosa PGPR2 root colonization

**Figure 2:**
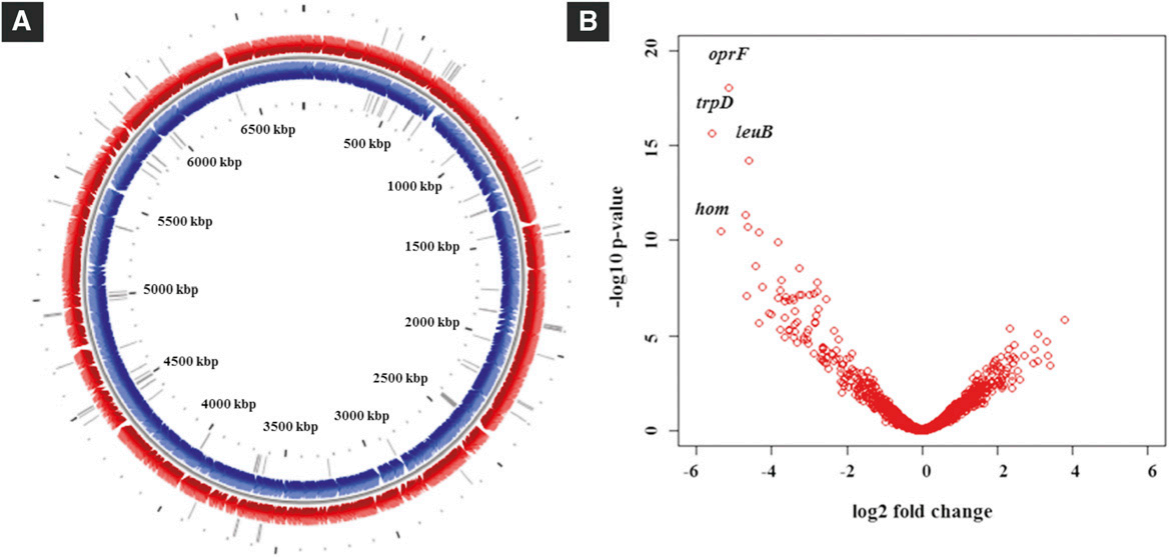
Mutants affected in colonization of corn roots. (A) Genome map showing transposon insertion sites of conditionally essential genes in *P. aeruginosa* PGPR2 genome. The outer circle represents forward strand (red), the inner circle represent reverse strand (blue), and the gray bars depict the transposon insertion sites. Circular plot was generated using the CG view tool (Stothard and Wishart 2005). (B) Volcano plot representing the genes responsible for fitness of PGPR2 in the corn rhizosphere. The fitness genes with highest significance are highlighted with name designations.

**Figure 3:**
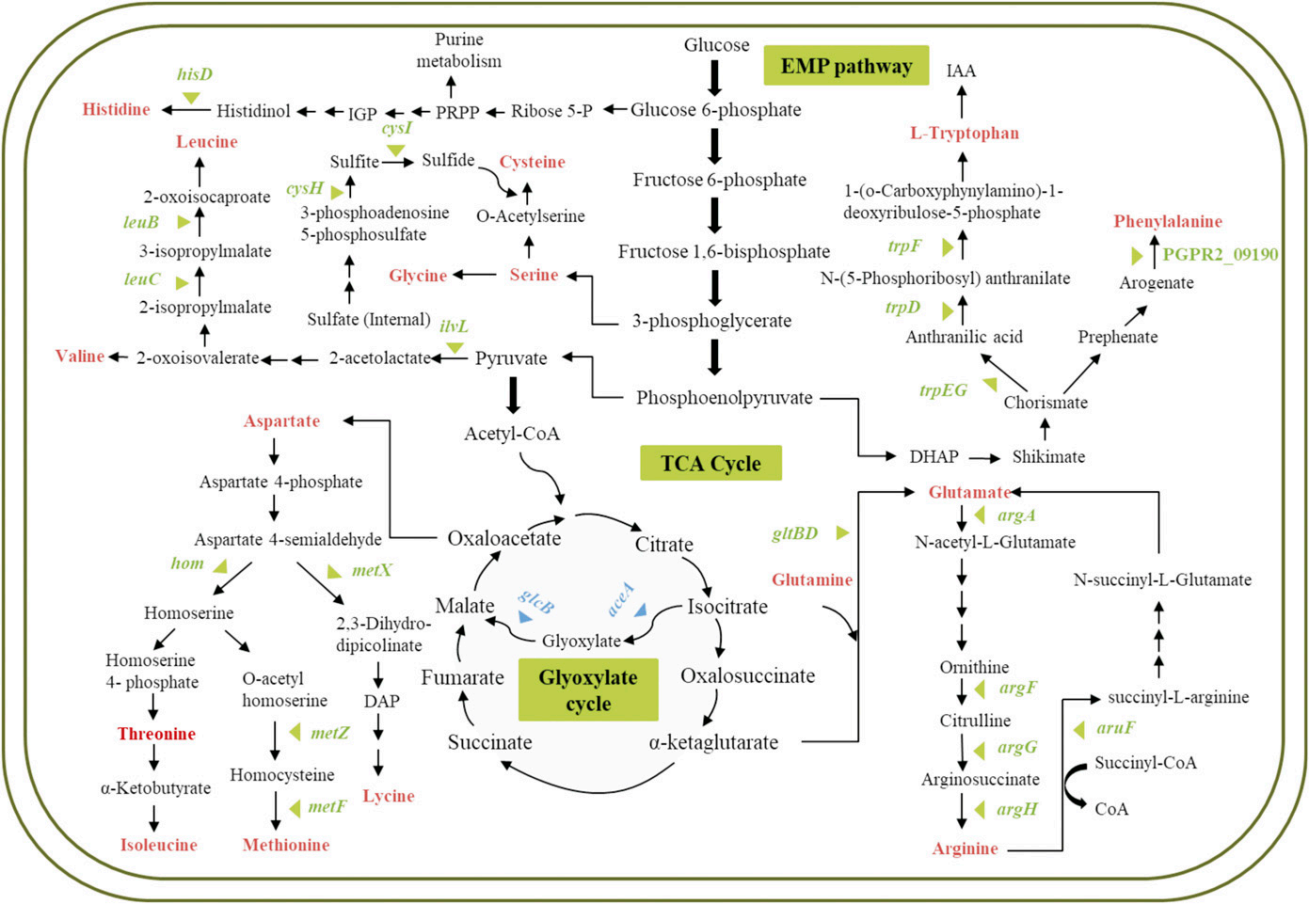
Essential genes involved in metabolic pathways (carbon and amino acid metabolism) responsible for fitness of *P*. *aeruginosa* PGPR2 in the corn root. Genes significantly (q < 0.05) influenced fitness during root colonization are marked with triangles; blue triangles indicate carbon metabolism and green triangles indicate amino acid metabolism.

Genes essential for fitness of PGPR2 during root colonization were identified by comparing the number of reads for each insertion site in the output pool to the number of reads in the input pool. Mutants with transposon insertions in 108 genes were underrepresented in the output pool with a minimum two-fold change (log_2_ < -1 with an adjusted p-value < 0.05), and were therefore considered to contain insertions in genes essential for fitness during root colonization (Figure 2A and 2B) (Table S3). We classified the fitness genes based on the Cluster of Orthologous Group (COG) annotation system (Fig. S5). Most of these genes encoded functions associated with amino acid transport and metabolism, energy production and conservation, and coenzyme transport and metabolism. The genes involved in metabolism responsible for the fitness of *P*. *aeruginosa* PGPR2 during root colonization are shown schematically in Figure 3. Other genes implicated in root colonization were associated with cell motility, cell wall/membrane/envelope biogenesis, signal transduction, and stress adaptation.

#### Metabolism:

Functional categorization revealed that approximately 40% of the identified fitness genes were involved in metabolism. Transposon insertions in 25 genes involved in amino acid utilization were significantly underrepresented in output population (Table 2). We found transposon insertions in four genes involved in tryptophan biosynthesis that exhibited 11- to 48-fold reductions in fitness (Table 2). The transposon insertion in a gene involved in the metabolism of aspartate resulted in a 41-fold reduction. Similarly, transposon insertions in genes involved in arginine and methionine biosynthesis pathways significantly reduced the fitness of PGPR2 from 13.3- to 4.4-fold during root colonization. Similarly, transposon insertions in the genes involved in the metabolism of leucine, histidine, isoleucine, glutamate, and cysteine reduced fitness from 24.7- to 3.5-fold.

Transposon insertions in carbohydrate utilization genes also displayed reduced fitness during root colonization (Fig. S5). Genes involved in glyoxylate cycle, energy production and cytochrome biosynthesis were essential for root colonization (Table 2). Transposon insertions in two signature genes of the glyoxylate cycle, isocitrate lyase and malate synthase, were underrepresented in the output population with the fold-change of -7.3 and -6.8 (Table 2). Of the fourteen genes comprising *neu* operon, our screen found that transposon insertion in seven genes (*nuoH*, *nuoB*, *nuoK*, *nuoE*, *nuoN*, *nuoM* and *nuoG*) led to a 10.4- to 4.4-fold reduction in fitness (Table 2). Three genes involved in cytochrome biosynthesis, encoding cytochrome C assembly protein, cytochrome B and cytochrome oxidase subunit I were significantly underrepresented in the output pool from corn roots. The mutants were impaired in colonization and displayed a fitness reduced from 4.5- to 3.0-fold (Table 2).

Besides, genes involved in purine-, fatty acid- and vitamin metabolisms were found to be essential for the root colonization. Transposon insertions in three genes involved in purine biosynthesis (*purF* coding for amidophosphoribosyl transferase, *purL* coding for phosphoribosyl formylglycinamidine synthase, and *purM* coding for phosphoribosyl aminoimidazole synthetase) were significantly underrepresented in the output pool of mutants. Mutants in these genes were significantly hampered in root colonization ability, reduced 8.4- to 10.5-fold (Table 2). Transposon insertions in two genes involved in fatty acid metabolism coding for acyl-CoA dehydrogenase and enoyl-CoA hydratase reduced fitness on roots 13.5- to 3.7- fold (Table 2). We identified five genes involved in the metabolism of vitamins when disrupted displayed significantly reduced fitness. Mutants in *cbiA*, *thiG*, *bioB* and *pdxA* genes, which are involved in the synthesis of water-soluble vitamins, showed reduced fitness from 4.8- to 5.2-fold (Table 2).

#### Motility and adhesion:

Transposon insertion in two genes involved in flagellar biosynthesis (*flhF* that code for flagellar biosynthesis regulator and *flgD* coding for flagellar basal body rod modification protein) were significantly underrepresented in the output population (Table 2). Disruption of these genes resulted in reduced fitness of 8.1- to 3.7- fold. These genes play a crucial role in flagella driven swimming motility, which is a prerequisite for root colonization (Allard-Massicotte *et al.*, 2016). Our screen identified a gene coding for fimbrial subunit *cupA*4 which is crucial for adhesion of bacteria to root when disrupted significantly reduced the fitness to 6.4- fold.

#### Stress response and detoxification:

Three genes, *algU*, *gshA*, and *gshB*, involved in stress responses, were underrepresented in the output population with reductions between 3.4- and 16.8-fold (Table 2). Transposon insertions in genes involved in the biosynthesis of glucans were significantly underrepresented in our screen with fold-changes of -12.5 and -9.2 (Table 2). These genes code for a periplasmic glucan biosynthesis protein (MdoG) and glucosyltransferase (MdoH) which are crucial for osmotic stress tolerance. A transposon insertion in *clpX*, which codes for ATPase subunit of the Clp protease (PGPR2_18895), significantly reduced fitness *in vivo* with the fold-change of 14 which is essential to degrade misfolded proteins and regulatory proteins to cope with stress conditions.

#### Signal transduction:

Transposon insertions in eight genes encoding sensor kinases and transcriptional regulators displayed reduced fitness. Transposon insertions in five genes involved in two-component systems (TCS) decreased fitness from 4.6- to 10.8-fold (Table 2). We also identified that the transposon insertion in a gene encoding a hybrid sensor kinase RetS, which is a regulator of exopolysaccharide and type III secretion system, reduced the fitness to 9.5 fold. Insertions in genes associated with the ArsC (PGPR2_08335) and MarR (PGPR2_12420) transcriptional regulators resulted in significantly reduced fitness by 8.9- and 5.8- fold (Table 2).

#### Transporters:

Transposon insertion in four ABC transporters (PGPR2_29805, PGPR2_12725, PGPR2_25320 and PGPR2_29810) reduced colonization fitness with fold-changes from 6.2- to 3.0- (Table 2). These genes code for an ABC transporter permease, multidrug efflux pump, and VacJ ABC transporter. Disruption of two genes coding for outer membrane proteins OprF and OprD resulted in reduced fitness with the fold-change of 35 and 3.3, respectively (Table 2).

#### Miscellaneous genes:

We also identified several other genes that might play significant roles in root colonization. Transposon insertions in genes involved in genetic rearrangement and horizontal gene transfer significantly reduced fitness. Genes involved in these processes such as *ruvB* encoding a DNA helicase, *parA*, encoding a chromosome-partitioning gene, and an insertion sequence *ISP*pu14 transposase were found to be important for root colonization by PGPR2. Disruption of these genes resulted in decreased fitness with the fold-change of 4.5- to 19-fold (Table 2). Besides the genes with known functions, we also identified five hypothetical proteins. Transposon insertions in these genes significantly reduced the fitness from 12.4- to 2.6- fold.

### Validation of root colonization by the mutants of P. aeruginosa PGPR2

**Figure 4:**
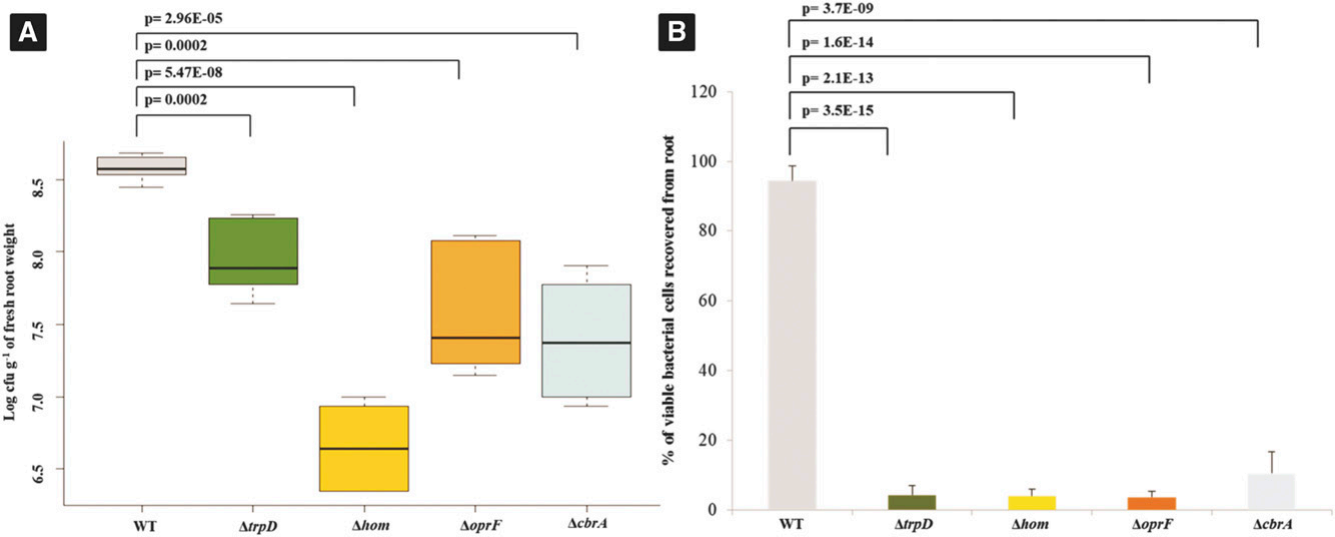
Validation of colonization mutants identified by INSeq screen. (A) Corn root colonization by wild type *P. aeruginosa* PGPR2 and its knockout deletion mutants. ∼4 × 10^6^ CFU ml^-1^ of wild type *P. aeruginosa* PGPR2 and each of the mutant strains (∆*trpD*::Gm^R^, ∆*hom*::Gm^R^, ∆*oprF*::Gm^R^ and ∆*cbrA*::Gm^R^) were inoculated individually and bacterial populations were determined seven days post inoculation. The colonization experiment was performed thrice independently with five replicates within each experiment. (B) Competitive corn root colonization by wild-type *P. aeruginosa* PGPR2 and its deletion mutants. 1:1 mixtures of wild type and one of the mutants were inoculated; the competitive colonizing ability was expressed as percentage of viable bacterial cells recovered from root.

To validate the INSeq experimental results, we constructed four isogenic mutants and tested for their ability to colonize corn roots both individually and in competition with the parent strain. We selected four genes, which showed the highest negative fold-changes (*trpD*, -48; *hom*, -41; *oprF*, -35; and *cbrA*, -11). All four knock-out mutants showed poor root-colonizing ability when tested either individually or when co-inoculated with the wild-type strain (Figure 4), thereby validating the INSeq results.

## Discussion

Root colonization is a dynamic process involving interactions between a microorganism and its plant host. *P. aeruginosa* PGPR2 is a plant growth-promoting bacterium and an efficient root colonizer (Illakkiam *et al.*, 2013; 2014). In this study, an INSeq mutant screen identified 108 genes essential for corn root colonization by *P. aeruginosa* PGPR2. Recently, Cole *et al.*, (2017) reported that 115 genes of *Pseudomonas simiae* are essential during root colonization of *Arabidopsis thaliana* including five (*cbrA*, *flhF*, *flgD*, *bioB*, and *parA*) identified in our study of *P. aeruginosa* PGPR2. *cbrA* encodes a sensor kinase that is part of the two-component system involved in assimilation of multiple carbon and nitrogen sources (Nishijyo *et al.*, 2001). *flhF* and *flgD* encode flagellar proteins required for motility and chemotaxis toward plant roots (Allard-Massicotte *et al.*, 2016). *bioB* is responsible for water-soluble vitamin biosynthesis. *parA* is required for normal partitioning of plasmids and the chromosome to daughter cells before cell division (Streit *et al.*, 1996). Secondary validation of the selected mutants indicated that most or all of the mutations identified are responsible for the observed phenotypes (Figure 4).

Most fitness genes we identified are involved in amino acid and carbon metabolism, possibly playing a role in utilization of metabolites from the root or exudate. For example, we found four genes (*trpD*, *trpG*, *trpE*, and *trpF*) involved in biosynthesis of tryptophan, which is used by bacteria as a building block in synthesis of siderophores, indole-3-acetic acid (IAA), signaling molecules, and disease-suppressive compounds, all of which are essential for root colonization (Matthijs *et al.*, 2004; Farrow and Pesci 2007; Balibar and Walsh 2006; and Roca *et al.*, 2012). Similarly, insertions in arginine biosynthesis genes such as *argA*, *argF*, *argG*, and *argH* in PGPR2 reduced fitness during corn root colonization. Previously, *P. fluorescens* WCS365 mutants auxotrophic for the arginine were reported to have diminished root-colonizing ability (Simons *et al.*, 1997) and insertions in genes involved in cysteine and leucine biosynthesis *leuB* reduced fitness of PGPR2 during root colonization.

Genes involved in the glyoxylate cycle, energy production and cytochrome biosynthesis were essential for root colonization of PGPR2, which is consistent with the known importance of carbon metabolism in the rhizosphere (Lugtenberg *et al.*, 2001). Notably, seven (*nuoH*, *nuoB*, *nuoK*, *nuoE*, *nuoN*, *nuoM*, *nuoG*) out of 14 genes in the *nuo* operon when disrupted significantly reduced the fitness of PGPR2 in root colonization. The bacterium requires energy generated by oxidative phosphorylation for motility and attachment to the root surface. The NADH dehydrogenase I (encoded by the *nuo* operon) provide electrons from NADH to quinones, an essential component in the electron transport chain (Efremov *et al.*, 2010). Also, NADH dehydrogenase I is involved in the generation of proton motive force needed for ATP synthesis, active transport of various nutrients across the membrane, and ATP-dependent flagellar rotation. A previous study showed that mutations in NADH dehydrogenase resulted in 100-fold impairment of colonization efficiency of *P. fluorescens* WCS365 (Camacho Carvaja *et al.*, 2002). Similarly, transposon insertions in *nuoL*, *nuoN*, and *nuoM* of a plant pathogen, *Dickeya dadantii* were essential for the infection on chicory leaf (Royet *et al.*, 2017).

Transposon insertions in three genes (*purF*, *purL* and, *purM*) involved in purine metabolism resulted in reduced fitness during root colonization. Recently, Duong *et al.* (2018) have reported that the *purF* and *purL* mutants of *Pantoea stewartii*, a phytopathogen, showed poor survival in the corn xylem. Gram-negative bacterial outer membrane protein (OMP) known as a porin for solute exchange have been implicated as surface receptors, in surface adherence, and in maintenance of structural integrity (Rollauer *et al.* 2015). Transposon insertions in *ompF* and *ompD* coding for outer membrane proteins significantly hampered fitness of PGPR2. Similarly, transposon insertions in *ompC* and *ompA* of *P. stewartii* resulted in reduced growth and virulence on corn (Duong *et al.*, 2018).

Besides the genes with known functions, we also identified five hypothetical proteins and ten genes categorized as having predicted general functions, are involved in root colonization (Fig. S5). Understanding the functions of these genes will provide novel insights into the pathways contributing to root colonization by PGPR2. The current work introduces genome-wide analysis to unravel understand beneficial plant-microbe interactions.

## Conclusion

**Figure 5:**
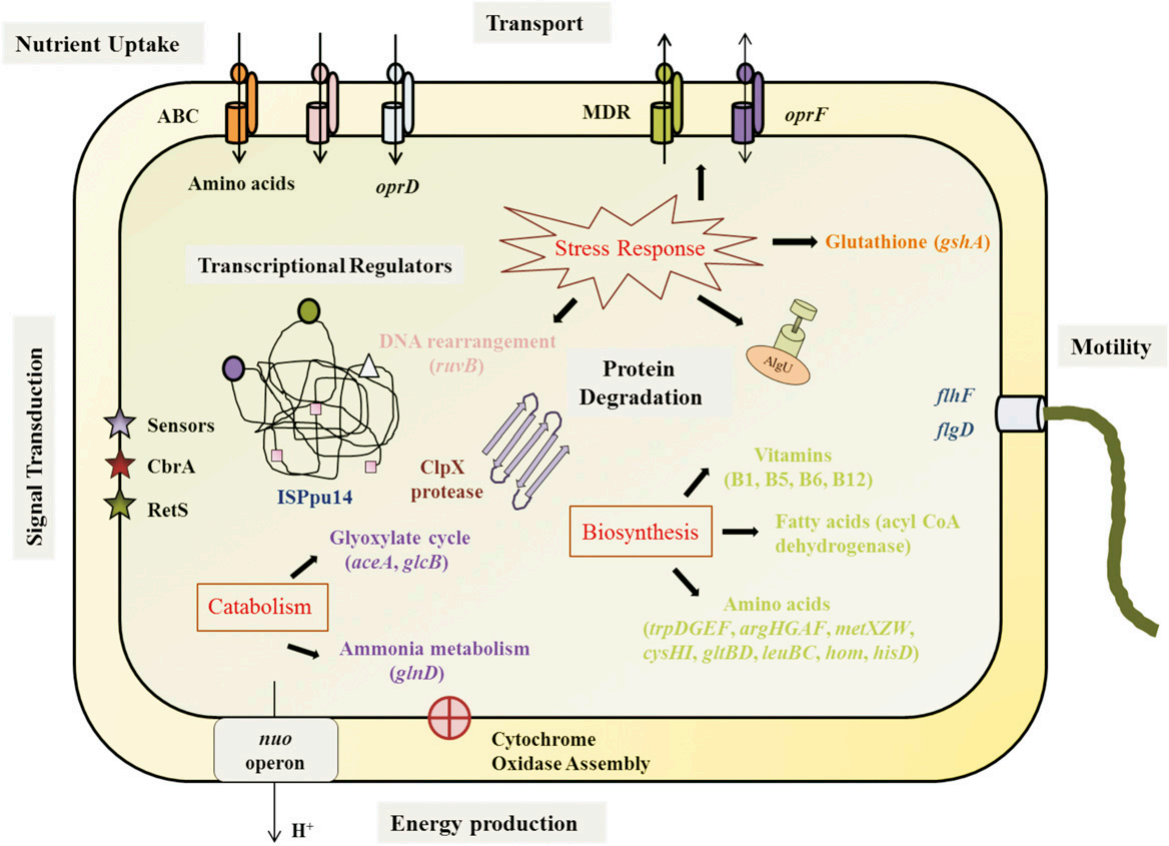
Integrated scheme showing the fitness genes identified in this study and their functions. See text for details

The transposon-insertion sequencing technique, INSeq, revealed 108 determinants of *P. aeruginosa* PGPR2 fitness during root colonization of corn. Many of the genes have functions previously shown to be important in root colonization, thus validating the INSeq approach. New functions were also identified as were genes with no homologs of known function. The key features required for root colonization identified in this study are integrated and shown in Figure 5. Further characterization of the fitness genes essential for root colonization may provide insights into novel pathways employed by bacteria that establish mutualistic relationships with their hosts. This study sheds light on the power of genome-wide approaches like INSeq in understanding complex mechanisms underlying plant-microbe interactions. Deciphering these relationships paves the way to promote these beneficial interactions for improving crop productivity in agriculture.
